# Supplementary material for: Individual Variabilities in Adipose Stem Cell Proliferation, Gene Expression and Responses to Lipopolysaccharide Stimulation
Source: Int J Mol Sci. 2022 Oct 19;23(20):12534. doi: 10.3390/ijms232012534 (PMC9604277; doi:10.3390/ijms232012534)
Supplement: Supplementary file 1 [file ijms-23-12534-s001.zip › ijms-1888786-supplementary.pdf]

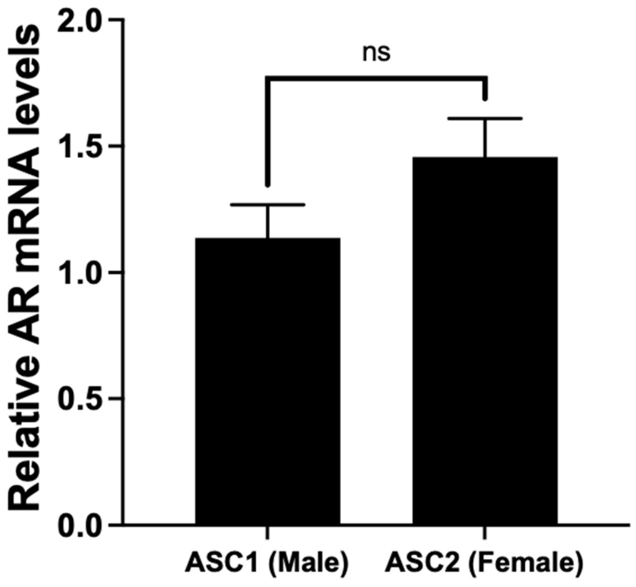

Figure S1. Comparison of androgen receptor (AR) expression levels in ASC.
ASCs were cultured as described in Materials and Methods. Cells were harvested, total RNA isolated and AR mRNA determined by RT-PCR. Results expressed as relative mRNA levels (mean+/-SD, n=3).

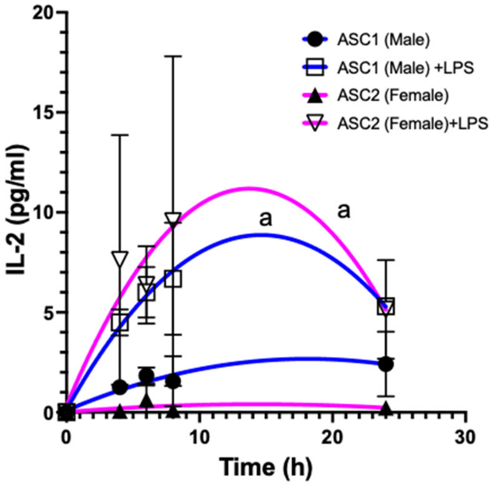

(a) IL-2

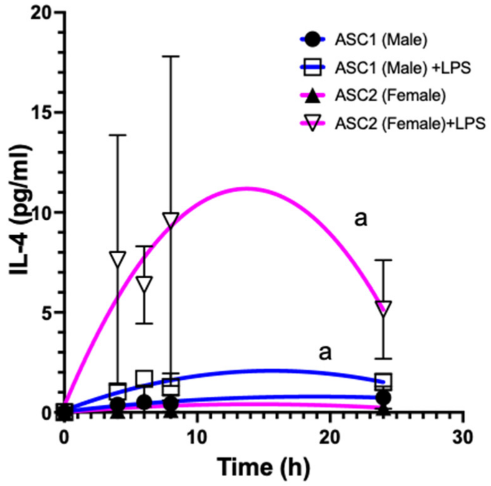

(b) IL-4

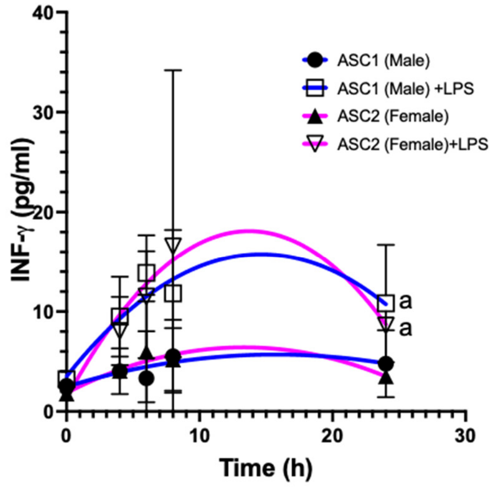

(c) INF-γ

Figure S2. Individual adipose stem cells (ASCs) differentially secreted IL-2, IL-4, INF-γ in response to LPS.

ASCs were cultured and treated with or without LPS (10 ng/ml), media were collected at different time points and selected cytokines protein levels were determined as described in Materials and Methods. Results expressed as pg/ml (mean+/-SD, n=4). (a) IL-2. (b) IL-4. (c) INF-γ. Curves with different letter indicate significantly different at $p<0.05$.
